# Towards Novel Spintronic Materials: Mg-Based *d*^0^-*d* Heusler (Nowotny–Juza) Compounds

**DOI:** 10.3390/mi16060674

**Published:** 2025-05-31

**Authors:** Kemal Özdoğan, Iosif Galanakis

**Affiliations:** 1 Department of Physics, Yildiz Technical University, 34210 İstanbul, Turkey; kozdogan@yildiz.edu.tr; 2 Department of Materials Science, School of Natural Sciences, University of Patras, GR-26504 Patra, Greece

**Keywords:** Heusler compounds, Nowotny–Juza compounds, ab initio calculations, electronic structure, magnetic materials, Slater–Pauling rule

## Abstract

Heusler compounds and alloys constitute a burgeoning class of materials with exceptional properties, holding immense promise for advanced technologies. Electronic band structure calculations are instrumental in driving research in this field. Nowotny–Juza compounds are similar to Semi-Heusler compounds containing one instead of two transition metal atoms in their chemical formula. Recently, they have been widely referred to as “$p^{0}$-*d* or $d^{0}$-*d* Semi-Heusler compounds”. Building upon our previous studies on $p^{0}$-*d* or $d^{0}$-*d* Semi-Heusler compounds featuring Li or K, we now explore a new class of $d^{0}$-*d* compounds incorporating alkaline earth metals and more specifically Mg which is well-known to occupy all possible sites in Heusler compounds. These compounds, with the general formula Mg*Z*(Ga, Ge, or As), where *Z* is a transition metal, are investigated for their structural, electronic, and magnetic properties, specifically within the context of the three possible $C1_{b}$ structures including also the effect of tetragonalization which is shown not to affect the equilibrium cubic type. Our findings demonstrate that a significant number of these compounds exhibit magnetic behavior, with several displaying half-metallicity, making them highly attractive for spintronic applications. This research provides a crucial foundation for future experimental investigations into these promising materials.

## 1. Introduction

Nowotny and Sibert as well as Juza and Hund in the early fifties studied compounds having the chemical formula XYZ where one of the three chemical elements is a transition metal atom [1,2]. Magnesium argentite, MgAgAs, is the prototype compound, which crystallizes in a variety of lattice structures. On the other hand, Heusler compounds are mostly cubic intermetallic compounds containing at least two different transition metal atoms in their chemical formula (e.g., NiMnSb [3]). Due to the extended interest on Heusler compounds, several studies have appeared recently on Nowotny–Juza compounds assuming the cubic lattice of Heusler compounds (or a tetragonal analogue). This specific class of compounds has been named as “$p^{0}$-*d*” or “$d^{0}$-*d*” Heusler compounds which is the terminology adopted in the present article.

Fritz Heusler, a German metallurgist, made a significant breakthrough in the early 20th century while exploring ways to improve steel’s electrical conductivity [4,5]. He discovered Cu_2_MnAl, a novel compound with a unique face-centered cubic (f.c.c.) crystal structure, similar to prominent semiconductors like silicon and gallium arsenide. This discovery led to the identification of a broader class of materials known as Heusler compounds, characterized by diverse intermetallic compositions and exhibiting unique properties [6,7,8]. Heusler compounds are categorized into four main types: Semi-Heusler (e.g., NiMnSb), Full-Heusler (e.g., Co_2_MnSi), Inverse Heusler (e.g., Mn_2_CoSi), and Quaternary Heusler compounds (e.g., (CoFe) TiSi), each with distinct crystal structures and properties [7,9]. Some Heusler compounds exhibit ferromagnetism with high Curie temperatures. Recently, research has intensified on all-*d*-metal Heusler compounds, a subset composed entirely of transition metal atoms, due to their promising potential [10,11,12,13].

A key characteristic of Heusler compounds is half-metallicity [14]. Half-metallic ferromagnetic or ferrimagnetic materials display metallic conductivity for electrons with one spin orientation (majority spin) while behaving as semiconductors for electrons with the opposite spin (minority spin) [15]. This unique property leads to high spin polarization at the Fermi level, making them highly promising for spintronics and magnetoelectronics applications by enabling novel electronic device functionalities. Heusler compounds offer a significant advantage by combining their half-metallic nature with high Curie temperatures. This combination has generated considerable research interest due to their potential for practical applications [16,17,18,19,20].

First-principles calculations have emerged as an indispensable tool for predicting and understanding material properties, including novel phenomena like spin-gapless semiconductors and spin-filtering [21]. Extensive databases of magnetic Heusler compounds, compiled through first-principles calculations, now exist, providing valuable insights into the properties of hundreds of these materials [22,23,24,25,26,27,28]. These databases complement in-depth studies of specific Heusler compounds. In magnetic Semi-Heusler compounds (XYZ), the *X* element is usually a transition metal or rare earth element. However, substituting *X* with alkali or alkaline earth metals results in “$p^{0}$-*d*” or “$d^{0}$-*d*” Heusler compounds. The “$p^{0}$” and “$d^{0}$” designations reflect the electronic configurations of the substituted elements: $p^{0}$ for elements like Li and Be, and $d^{0}$ for elements like Na, Mg, K, Rb, Cs, Ca, Sr, and Ba.

Extensive research has been conducted on $d^{0}$-*d* and $p^{0}$-*d* Heusler compounds. Previous studies, including those by Damewood et al. [29] and Dehghan and Davatolhagh [30,31,32], have utilized first-principles calculations to investigate various compounds and develop comprehensive databases. These databases, while valuable, lack detailed information on individual compounds. To address this gap, we conducted in-depth studies on Li*Z*Ga, Li*Z*Ge, K*Z*Ga, K*Z*Ge, K*Z*As, and K*Z*Se compounds, where *Z* is a 3*d* transition metal [33,34]. Our findings revealed unexpected structural variations and identified compounds exhibiting half-metallicity across multiple structural variants.

## 2. Materials and Methods

The present study focuses on the $d^{0}$-*d* Heusler compounds where *X* is Mg, an alkaline earth metal, and more specifically on the Mg*Z*Ga, Mg*Z*Ge, and Mg*Z*As compounds, where *Z* varies across the 3*d* transition metal series (Sc to Ni). The reasoning behind the choice of Mg is due to its versatile character in Heusler compounds. It is one of the few chemical elements which can occupy all equivalent sites in Heusler compounds [9]. This systematic approach allows us to examine the influence of both the transition metal atom and the metalloid element (Ga, Ge, and As) on the properties of these compounds.

For our first-principles calculations, we employed the full-potential nonorthogonal local-orbital minimum-basis band structure method (FPLO) (version FPLO18.00-52) [35,36] in conjunction with the Perdew–Burke–Ernzerhof (PBE) parameterization of the generalized gradient approximation (GGA) [37]. The computational details are identical to those described in reference [33]. It is important to note that GGA-PBE has been shown to accurately reproduce the properties of half-metallic Heusler compounds, comparable to more sophisticated meta-GGA functionals [38].

## 3. Results and Discussion

### 3.1. Structural Properties

**Figure 1:**
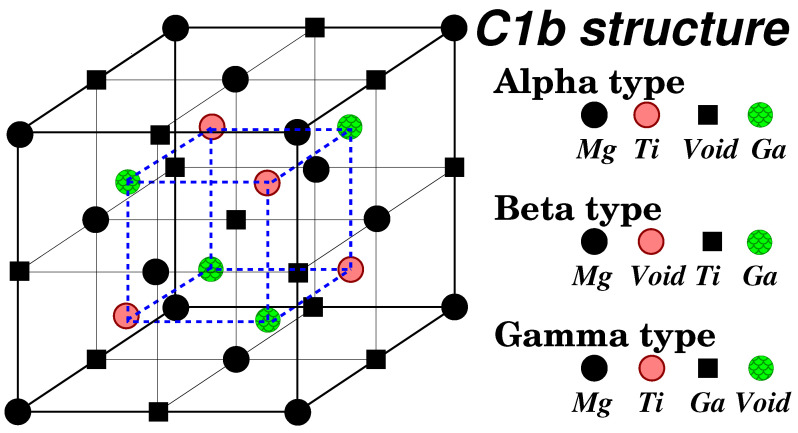
Schematic representation of the three possible types of the $C1_{b}$ structure adopted by the Semi-Heusler compounds using MgTiGa as the prototype. The black spheres, pink spheres, black squares, and green spheres are widely called A, B, C, and D sites, respectively. The large cube in the figure contains exactly four primitive unit cells.

Semi-Heusler compounds, such as those investigated in this study, typically crystallize in the $C1_{b}$ cubic lattice structure [6,7]. In Figure 1, the three possible variants of the $C1_{b}$ structure taking MgTiGa as the prototype are depicted. This structure comprises four sites, with one site remaining unoccupied. The arrangement of atoms within these sites gives rise to three distinct structural variants: the α, β, and γ types. These types differ primarily in the chemical nature of neighboring atoms, significantly influencing the orbital interactions between them. Consequently, the physical properties of a given compound can vary considerably across these different structural types, despite maintaining overall tetrahedral symmetry. Here, we should note that, as discussed above, the Nowotny–Juza compounds usually crystallize in a variety of structures and not the cubic ones assumed in this study. For example, MnNiGe crystallizes in the so-called TiNiSi structure [39], MgMnGe compound crystallizes in the PbClF lattice structure [40], MgFeGe crystallizes in the so-called anti-PbClF structure [41], and MnCoGe in a layered structure [42]. One expects that the cubic structure of Heusler compounds can be stabilized in the form of a 2D structure (films, multilayers, etc.) using modern growth techniques.

Table 1 presents the calculated equilibrium lattice parameters for all compounds analyzed in this study. These values were determined by minimizing the total energy across a range of lattice constants. The calculated lattice constants for Mg-based compounds range from approximately 5.8 Å to 6.7 Å. These values align with typical lattice constants observed in other Heusler compounds and semiconductors [6,7], an important consideration for applications involving multilayer heterostructures.

General trends in lattice constants are observed. Within a series, the lattice constants generally decrease as the atomic radii of the constituent elements decrease. For example, the lattice constants tend to decrease from Ga to As within a group, and from Sc to V across the 3*d* transition metal series. These trends are consistent with observations in Li-based $p^{0}$-*d* and K-based $d^{0}$-*d* Heusler compounds [33,34]. Comparing lattice constants across different types (α, β, and γ) reveals distinct trends. In contrast to Li-based compounds where the α type typically exhibits the smallest lattice constant [33], the α type in Mg-based compounds generally has the largest lattice constant, similarly to the K-based Heuslers [34], with exceptions observed for some Sc compounds and MgTiGa. When comparing the lattice constants between the the γ and β types, there is no clear trend: the β type has a smaller lattice constant for lighter transition metals, while the γ type has a smaller lattice constant for heavier transition metals. The factors influencing these variations in lattice constants are complex. Both chemical composition and magnetic properties play significant roles, as magnetic and non-magnetic types of the same compound often exhibit different equilibrium lattice constants [43].

Table 1 also presents the calculated total energy differences between the three types (α, β, and γ) at their respective equilibrium lattice constants. These energies are reported per formula unit in units of eV. A negative value indicates that the first type listed is more stable than the second type. In our previous studies, the stability of different types varied. For Li- based compounds [33], the β type was generally the most stable, with the γ type becoming more stable for late transition metals. For K-based compounds [34] the observed trend was the same but now the energy differences between the α type and the β/γ types were significantly larger, often exceeding 2 eV. The stability trends observed in Mg-based compounds exhibit similarities to both Li- and K-based systems. The α type is consistently the least stable, except in the case of MgScGa. The β type is generally the most stable, with the exception of compounds containing heavier transition metals, where the γ type becomes the most stable. In these two lattice types, each Mg atom has as nearest neighbors four vacant sites (Voids in Figure 1) and four *Z* or four Ga/Ge/As atoms for the β and γ types, respectively. As we move from the lighter to the heavier transition metal atoms the energy position of the *Z* valence orbitals changes and this may explain the fact that the γ type becomes more stable.

**Figure 2:**
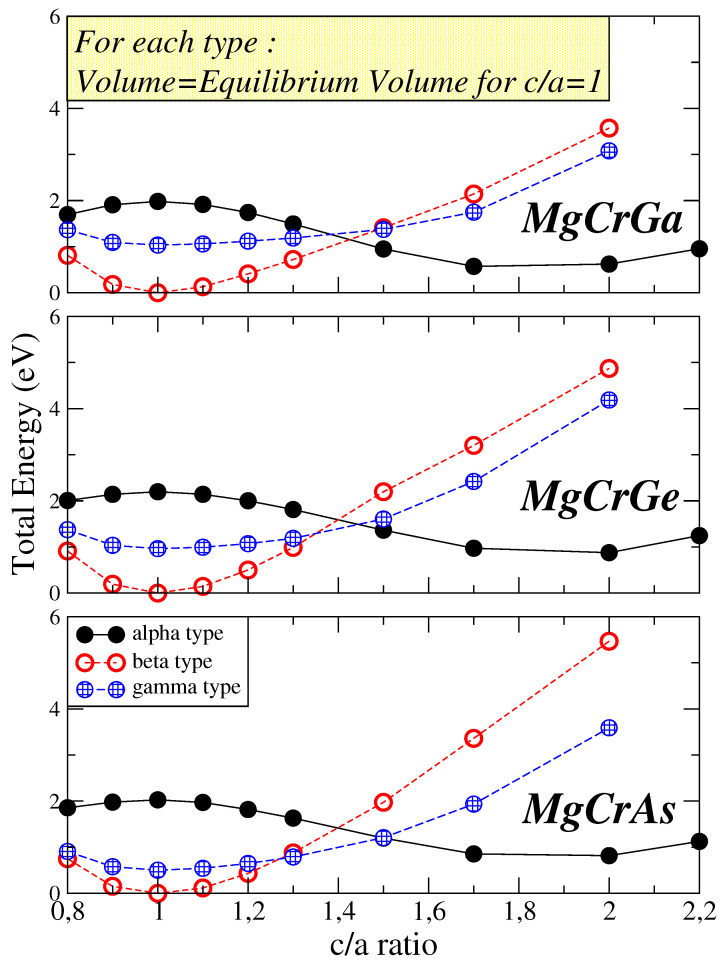
Calculated total energy as a function of the c/a ratio for the MgCrGa, MgCrGe and MgCrAs compounds assuming all three types. For each type and compound the volume of the unit cell was kept constant and equal to the equilibrium cubic solution presented in Table 1 upon the c/a variation.

Recently, in reference [44], they argued that for the K-based $d^{0}-d$ Heusler compounds the alpha type presents tetragonalization with a large c/a ratio which becomes the ground state. To test the effect of tetragonalization we kept the unit cell volume equal to the equilibrium cubic one and varied the c/a ratio. For example, in Figure 2, we present for the MnCr (Ga, Ge, and As) compounds the variation in total energy with respect to the c/a ratio for all three types. For all three compounds, the β and γ types have there minimum at the cubic type (c/a=1). On the contrary and in accordance with the results in reference [44] the minimum for the α type corresponds to a tetragonal type with a very high c/a ratio which is about 1.8 for MgCrGa and around 2 for the other two compounds. For all three compounds under study, the global equilibrium is the β cubic type. As we move from Ga to As, a change occurs between the relative position of the α and γ types. For MgCrGa the tetragonal α type is more stable than the cubic γ type. For MgCrGe both correspond almost to identical energy values and are degenerate, while for the heavier MgCrAs the cubic γ type is more stable. Since the magnetic properties do not vary drastically upon tetragonalization, we will focus only on the cubic case also for the α type when discussing the electronic and magnetic properties of the compounds in the next sections.

### 3.2. Electronic Properties

**Figure 3:**
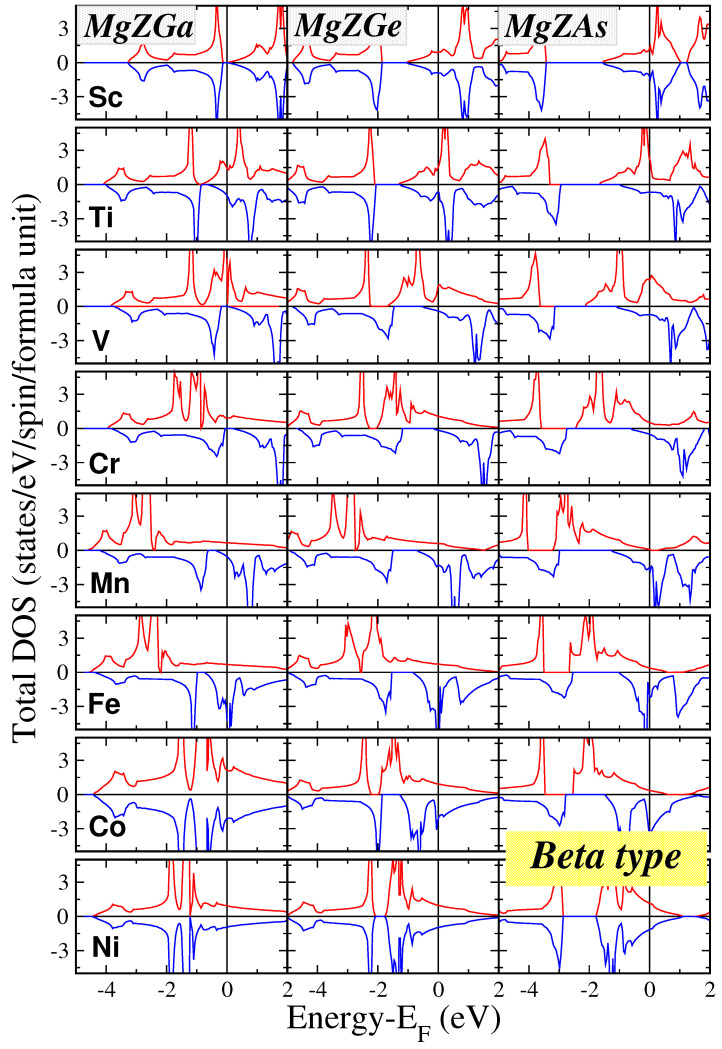
Total density of states (DOS) for the Mg*Z*Ga, Mg*Z*Ge, and Mg*Z*As compounds assuming the beta type. The Fermi level is set as the zero energy point. Positive and negative DOS values represent spin-up and spin-down electrons, respectively.

Electronic band structure calculations were performed for all 24 compounds across three types at their respective equilibrium lattice constants. The resulting total density of states (DOS) per formula unit (f.u.) is presented for Mg*Z*Ga, Mg*Z*Ge, and Mg*Z*As compounds in Figure 3 assuming the β type which is the most stable in most cases. The DOS of the γ type is very similar to the one of the β type as discussed in the next paragraph. To enable direct comparison, the DOS plots are standardized within an energy window of −4.5 eV to 2 eV with a vertical axis range of −4.5 to 4.5 states per eV per spin per f.u. A small contribution to the DOS below this energy window arises from the *s* states of Mg atoms.

The distinct DOS profiles observed for the α type compared to the β and γ types correlate with differences in their crystal structures. In the β and γ types, the proximity of *Z* and Ga/Ge/As atoms as nearest neighbors results in strong bonding interactions, leading to similar DOS characteristics. Conversely, in the α type, these atoms are next-nearest neighbors, resulting in weaker interactions and a significantly different DOS shape. This weaker bonding in the α type is consistent with its lower stability, as evidenced in Table 1.

Unlike Li compounds (reference [33]), but similarly to K-based Semi-Heusler compounds (reference [34]), Mg compounds predominantly exhibit magnetic behavior, as summarized in Table 2 and further elaborated upon below. Only a few Mg compounds are non-magnetic metals or semiconductors. These typically involve Sc (the lightest transition metal) or Ni (the heaviest transition metal), where the 3*d* electron states are nearly or fully occupied. Many Mg-based compounds exhibit perfect or near half-metallic properties, displaying metallic behavior for spin-up electrons and semiconducting behavior for spin-down electrons across all three types. Notable examples include MgVGa, MgCrGa, MgVGe, MgCrGe, and MgCrAs. In near half-metallic compounds, the total spin magnetic moment is very close to the ideal integer value, and the Fermi level lies marginally within or just outside the spin-down energy gap, as will be discussed further in the next subsection.

**Figure 4:**
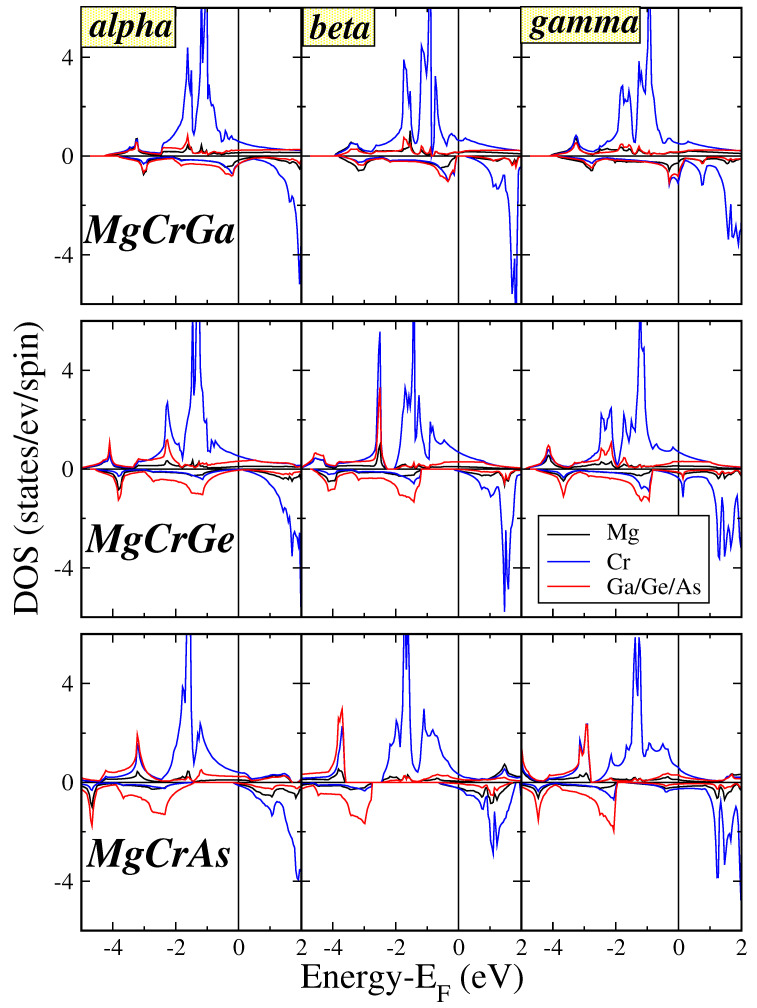
Atom -resolved DOS for the MgCrGa, MgCrGe, and MgCrAs compounds assuming all three types. Details as in Figure 3.

The observed DOS characteristics closely resemble those reported for Li and K compounds in references [33,34]. Mg atoms contribute a *s*-character band low in energy or please delete this phrase. in the energy window presented in Figure 3 and Figure 4 The primary contribution to the DOS arises from strong *p*-*d* hybridization between the *p* orbitals of Ga/Ge/As and the *d* orbitals of the transition metal atoms. Within the tetrahedral symmetry, the *d* orbitals split into two groups:

1. $t_{2g}$: This triply degenerate set hybridizes with the *p* orbitals of Ga/Ge/As, forming delocalized bonding states across both the transition metal and the pnictogen atoms.
2. $e_{g}$: This doubly degenerate set remains localized on the transition metal atom due to symmetry restrictions and does not participate in significant hybridization.

To make our argument clear the atom-resolved DOS for the MgCrGa, MgCrGe, and MgCrAs compounds for all three types is presented in Figure 4. All cases under study are half- or near half-metallic metallic as stated above. The occupied spin-up valence bands are dominated by the *d*-DOS of the Cr atoms above −2 eV. Below this value, there is a strong contribution of the Ga/Ge/As valence *p*-states. In the spin-down electronic band structure there is a gap separating occupied and unoccupied bands. The spin-down conduction bands have a Cr *d*-character while the occupied valence spin-down bands below the Fermi level have mainly a Ga/Ge/As *p*-character with a Cr *d*-admixture which is sizable for MgCrGa but almost vanishes for MgCrAs. These findings support our statements and show that the spin-down energy gap arises due to p−d hybridization between the Ga/Ge/As and the Cr atoms.

### 3.3. Magnetic Properties

The majority of compounds in this study exhibit a magnetic behavior. Table 2 presents the calculated atomic and total spin magnetic moments at equilibrium lattice constants. Non-magnetic compounds, are classified as metals with the sole exception of MgScGa in the β type which is a gapless semiconductor. Alkaline earth atoms generally contribute weakly to the total spin magnetic moment. The spin magnetic moments are primarily localized on the transition metal atoms. However, due to strong hybridization between the Ga/Ge/As *p* states and the transition metal -$t_{2g}$ states, significantly induced spin magnetic moments are also present on the Ga/Ge/As atoms. These induced moments in the case of Ga and Ge are antiparallel to the spin magnetic moments of the transition metal atoms in all cases, while in the case of the As compounds they are parallel in the case where *Z* is a late transition metal atom.

**Figure 5:**
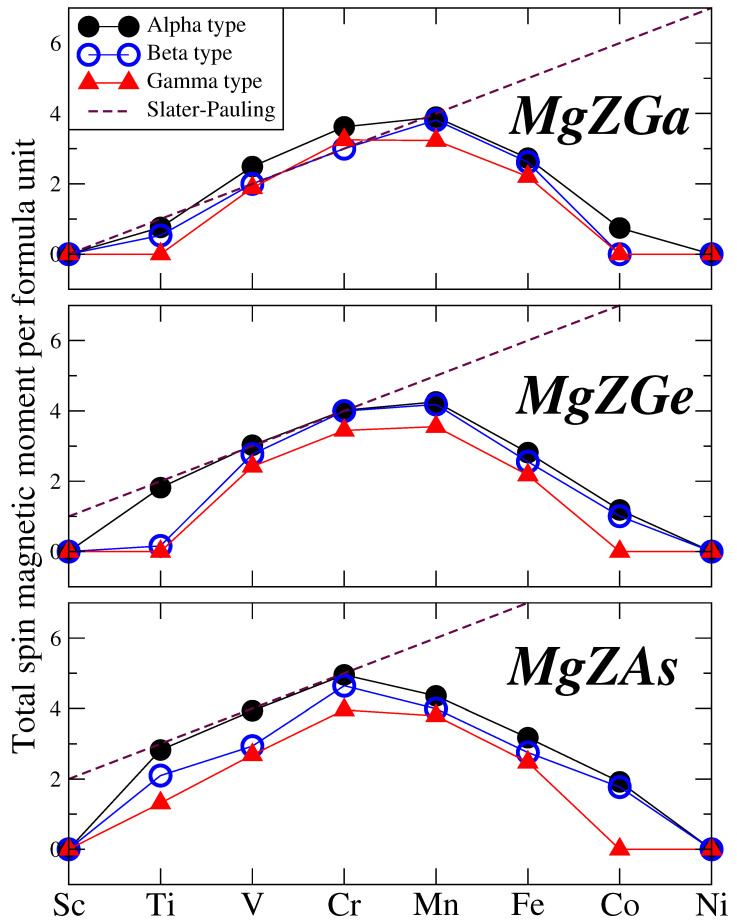
Total spin magnetic per formula unit in $\mu_{B}$ units as a function of the *Z* chemical element (Z= Sc → Ni) for all three studied families of Mg-based compounds. The black dashed lines represent the ideal Slater Pauling rules for half-metallicity: $M_{t}$ = $Z_{t}$ − 8.

We now turn our attention to the total spin magnetic moment ($M_{t}$) per formula unit. Table 2 also provides the total number of valence electrons per unit cell ($Z_{t}$) and the total spin magnetic moment predicted by the Slater–Pauling rule, which will be discussed below. We should note here that the initial work by Slater and Pauling focused on the transition metal elements and binary compounds, but in the literature with the general term “Slater–Pauling rule” one addresses all expressions connecting the total spin magnetic moment to the number of valence electrons in half-metallic compounds. All spin magnetic moments are reported in units of $\mu_{B}$. Figure 5 illustrates the total spin magnetic moment as a function of the transition metal atomic number for all three families of compounds and across all three types. The black dashed line represents the Slater–Pauling rule. Compounds exhibiting integer values of their total spin magnetic moment, aligning with the Slater–Pauling rule, are identified as half-metallic ferromagnets, consistent with their previously discussed total density of states. Similarly to the Li-based $p^{0}$-*d* compounds (reference [33]) and unlike the K-based $d^{0}$-*d* compounds (reference [34]), half-metallicity is not common in Mg-based compounds. When the number of valence electrons ($Z_{t}$), as listed in Table 2 reaches or exceeds 13, achieving half metallicity would require a total spin magnetic moment of 5 $\mu_{B}$ or higher. Such high magnetic moments are energetically unfavorable, leading to the loss of half-metallicity, and the compounds remain simply magnetic. MgVGa, MgCrGa, MgCrGe, and MgCrAs compounds exhibiting half-metallic or near half-metallic behavior across all three types are particularly promising for spintronic and magnetoelectronic applications.

The origin of half-metallicity has been discussed in the previous section. For half-metallic compounds the spin-down band structure exhibits exactly four fully occupied states: a low-energy Mg *s* valence state and the triple degenerate Ga/Ge/As *p*-*Z* $t_{2g}$ bonding hybrids (there are also three antibonding hybrids above the Fermi level). The total spin magnetic moment corresponds to the number of uncompensated spins. For a compound to exhibit half-metallicity, the total spin magnetic moment must adhere to the Slater–Pauling rule: $M_{t}=Z_{t}-2\times 4=Z_{t}-8$, where $Z_{t}$ is the total number of valence electrons per unit cell.

## 4. Summary and Conclusions

Half-metallic Semi-Heusler compounds are of significant interest due to their potential applications in spintronics. These compounds exhibit three distinct crystal structures within the $C1_{b}$ lattice: α, β, and γ, each with a unique atomic arrangement. This study employs first-principles electronic band structure calculations to investigate the structural, electronic, and magnetic properties of Mg*Z*(Ga, Ge, or As) $d^{0}$-*d* Heusler compounds, where *Z* represents transition metals from Sc to Ni, across all three $C1_{b}$ types. Our findings indicate that the β and γ types are generally more stable than the α type. This stability trend is attributed to variations in local atomic environments and crystal symmetries across the different types. Tetragonalization occurs only in the case of the α type with a large c/a ratio but this does not alter the global energy minimum and the equilibrium type. The majority of compounds exhibit magnetic behavior, with a few displaying half-metallicity, consistent with the Slater–Pauling rule. Notably, MgTiGa, MgVGa, MgCrGa, MgMnGa, MgVGe, MgCrGe, and MgCrAs demonstrate perfect or near half-metallic ferrimagnetism at the equilibrium β type highlighting their potential for spintronic applications.

These results are expected to stimulate further experimental and theoretical investigations into these promising materials for advancements in spintronics and magnetoelectronics.

## Figures and Tables

**Table 1:** Equilibrium lattice constants are provided for all compounds in all three types (α, β, γ). Subsequent columns show the total energy difference between types, calculated using each type’s equilibrium lattice constant. The final two columns indicate the most and least stable types.

Compound	Lattice Constant *a* in Å			Energy Difference ΔE in eV			Most Stable	Least Stable
	α-Type	β-Type	γ-Type	$E_{\beta }-E_{\alpha }$	$E_{\beta }-E_{\gamma }$	$E_{\alpha }-E_{\gamma }$	Type	Type
MgScGa	6.47	6.49	6.64	−1.129	−1.532	−0.403	β	γ
MgTiGa	6.27	6.24	6.36	−1.275	−1.000	0.275	β	α
MgVGa	6.26	6.18	6.24	−1.186	−0.838	0.348	β	α
MgCrGa	6.30	6.17	6.23	−0.993	−0.520	0.473	β	α
MgMnGa	6.28	6.19	6.16	−1.012	−0.290	0.723	β	α
MgFeGa	6.14	6.04	6.03	−1.060	0.137	1.197	γ	α
MgCoGa	5.99	5.88	5.86	−1.123	0.443	1.566	γ	α
MgNiGa	6.02	5.93	5.90	−0.886	0.602	1.487	γ	α
MgScGe	6.39	6.41	6.55	−1.358	−1.203	0.155	β	α
MgTiGe	6.29	6.19	6.29	−1.482	−0.796	0.686	β	α
MgVGe	6.24	6.18	6.19	−1.321	−0.787	0.534	β	α
MgCrGe	6.27	6.22	6.17	−1.101	−0.483	0.618	β	α
MgMnGe	6.25	6.19	6.12	−1.174	−0.167	1.007	β	α
MgFeGe	6.12	6.01	5.95	−1.271	0.178	1.449	γ	α
MgCoGe	6.01	5.89	5.80	−1.266	0.521	1.787	γ	α
MgNiGe	6.00	5.92	5.84	−1.058	0.641	1.699	γ	α
MgScAs	6.47	6.37	6.57	−1.432	−0.668	0.764	β	α
MgTiAs	6.37	6.23	6.31	−1.584	−0.759	0.824	β	α
MgVAs	6.29	6.14	6.19	−1.194	−0.413	0.781	β	α
MgCrAs	6.34	6.26	6.19	−1.017	−0.253	0.764	β	α
MgMnAs	6.26	6.14	6.11	−1.163	−0.116	1.047	β	α
MgFeAs	6.15	6.01	5.94	−1.406	−0.021	1.385	β	α
MgCoAs	6.06	5.93	5.77	−1.227	0.360	1.587	γ	α
MgNiAs	5.99	5.91	5.81	−1.172	0.531	1.702	γ	α

**Table 2:** For Mg*Z*Ga, Mg*Z*Ge, and Mg*Z*As compounds, we present atom-resolved spin magnetic moments in units of Bohr magnetons ($\mu_{B}$). We also provide the total spin magnetic moment ($m^{total}$) per formula unit, which is equivalent to the value per unit cell. The last columns list the total number of valence electrons per unit cell ($Z_{t}$) and in parenthesis the ideal total spin magnetic moment ($m^{S-P}$) predicted by the Slater–Pauling rules. For non-magnetic compounds we only report their electronic character.

		Mg*Z*Ga					Mg*Z*Ge					Mg*Z*As				
*Z*		$m^{\mathrm{Mg}}$	$m^{Z}$	$m^{\mathrm{Ga}}$	$m^{\mathrm{total}}$	$Z_{t}$ ( $m^{SP}$ )	$m^{\mathrm{Mg}}$	$m^{Z}$	$m^{\mathrm{Ge}}$	$m^{\mathrm{total}}$	$Z_{t}$ ( $m^{SP}$ )	$m^{\mathrm{Mg}}$	$m^{Z}$	$m^{As}$	$m^{\mathrm{total}}$	$Z_{t}$ ( $m^{SP}$ )
Sc	α	Metal				8 (0)	Metal				9 (1)	Metal				10 (2)
	β	Gapless Semiconductor				8 (0)	Metal				9 (1)	Metal				10 (2)
	γ	Metal				8 (0)	Metal				9 (1)	Metal				10 (2)
Ti	α	0.05	0.83	−0.13	0.76	9 (1)	0.17	1.90	−0.25	1.82	10 (2)	0.44	2.56	−0.18	2.82	11 (3)
	β	−0.01	0.58	−0.05	0.53	9 (1)	0.00	0.18	−0.02	0.16	10 (2)	0.19	1.95	−0.05	2.10	11 (3)
	γ	Metal				9 (1)	Metal				10 (2)	0.09	1.37	−0.15	1.31	11 (3)
V	α	0.00	2.85	−0.37	2.49	10 (2)	0.12	3.30	−0.40	3.02	11 (3)	0.34	3.81	−0.21	3.94	12 (4)
	β	−0.11	2.41	−0.31	2.00	10 (2)	0.06	3.01	−0.30	2.77	11 (3)	0.08	3.00	−0.14	2.93	12 (4)
	γ	−0.06	2.21	−0.26	1.89	10 (2)	0.06	2.74	−0.38	2.42	11 (3)	0.08	2.90	−0.30	2.69	12 (4)
Cr	α	−0.14	4.21	−0.46	3.62	11 (3)	0.01	4.49	−0.48	4.02	12 (4)	0.23	4.88	−0.16	4.95	13 (5)
	β	−0.22	3.73	−0.51	3.00	11 (3)	0.06	4.32	−0.40	3.97	12 (4)	0.14	4.65	−0.15	4.65	13 (5)
	γ	−0.17	3.83	−0.41	3.25	11 (3)	0.03	3.92	−0.50	3.45	12 (4)	0.09	4.18	−0.32	3.96	13 (5)
Mn	α	−0.11	4.31	−0.31	3.90	12 (4)	0.01	4.41	−0.17	4.26	13 (5)	−0.02	4.35	0.03	4.36	14 (6)
	β	−0.11	4.23	−0.31	3.81	12 (4)	−0.06	4.32	−0.08	4.19	13 (5)	−0.18	4.10	0.07	4.0	14 (6)
	γ	−0.15	3.75	−0.38	3.23	12 (4)	−0.03	3.85	−0.27	3.55	13 (5)	−0.07	3.89	−0.03	3.79	14 (6)
Fe	α	−0.14	3.04	−0.17	2.73	13 (5)	−0.08	3.03	−0.13	2.81	14 (6)	−0.09	3.13	0.13	3.17	15 (7)
	β	−0.11	2.93	−0.20	2.62	13 (5)	−0.07	2.77	−0.15	2.56	14 (6)	−0.10	2.82	0.03	2.75	15 (7)
	γ	−0.12	2.54	−0.21	2.21	13 (5)	−0.06	2.40	−0.16	2.18	14 (6)	−0.07	2.53	0.02	2.48	15 (7)
Co	α	−0.09	0.95	−0.12	0.74	14 (6)	−0.05	1.40	−0.17	1.18	15 (7)	−0.01	1.86	0.08	1.92	16 (8)
	β	Metal				14 (6)	−0.01	1.05	−0.03	1.01	15 (7)	−0.05	1.69	0.14	1.77	16 (8)
	γ	Metal				14 (6)	Metal				15 (7)	Metal				16 (8)
Ni	α	Metal				15 (7)	Metal				16 (8)	Metal				17 (9)
	β	Metal				15 (7)	Metal				16 (8)	Metal				17 (9)
	γ	Metal				15 (7)	Metal				16 (8)	Metal				17 (9)

## Data Availability

The data presented in this study are available on request from the corresponding author.

## Abbreviations

The following abbreviations are used in this manuscript:

DOS	Density of States
f.u.	formula unit
FPLO	Full-potential nonorthogonal local-orbital minimum- basis band structure approach
GGA	Generalized gradient approximation
PBE	Perdew Burke Ernzerhof
